# Melatonin Increases Bone Mass around the Prostheses of OVX Rats by Ameliorating Mitochondrial Oxidative Stress via the SIRT3/SOD2 Signaling Pathway

**DOI:** 10.1155/2019/4019619

**Published:** 2019-04-11

**Authors:** Wei Zhou, Yu Liu, Jining Shen, Binqing Yu, Jiaxiang Bai, Jiayi Lin, Xiaobin Guo, Houyi Sun, Zhanghuan Chen, Huilin Yang, Yaozeng Xu, Dechun Geng

**Affiliations:** Department of Orthopedics, The First Affiliated Hospital of Soochow University, Suzhou 215006, China

## Abstract

Bone mass loss around prostheses is a major cause of implant failure, especially in postmenopausal osteoporosis patients. In osteoporosis, excess oxidative stress largely contributed abnormal bone remodeling. Melatonin, which is synthesized from the pineal gland, promotes osteoblast differentiation and bone formation and has effectively been used to combat oxidative stress. Thus, we determined if melatonin can inhibit oxidative stress to promote osteogenesis and improve bone mass around prostheses in osteoporosis. In this study, we observed that received melatonin at 50 mg/kg body weight significantly increased periprosthetic bone mass as well as implant fixation intensity in ovariectomized (OVX) rats. Meanwhile, it decreased the expression of oxidative stress markers (NAPDH oxidase 2 and cytochrome c) and enhanced expressing level of the formation markers of bones (alkaline phosphatase, osteocalcin, and osterix) around prostheses compared to that in the control group. Additionally, melatonin decreased hydrogen peroxide- (H_2_O_2_-) induced oxidative stress and restored the osteogenesis potential of MC3T3-E1 cells. Mechanistically, melatonin clearly increased mitochondrial sirtuin 3 (SIRT3) expression and decreased the ratio of acetylated superoxide dismutase 2 (AC-SOD2)/SOD2 compared to the H_2_O_2_ group. SIRT3 inhibition counteracted the protective effects of melatonin on oxidative stress and bone formation. Together, the results showed that melatonin ameliorated oxidative stress in mitochondrial via the SIRT3/SOD2 signaling pathway, thereby promoting osteogenesis, improving bone mass around the prostheses, and increasing initial stability. Thus, melatonin might be a suitable candidate to decrease the rate of implant failure and lengthen the lifespan of prostheses after total joint arthroplasty.

## 1. Introduction

Total joint arthroplasty (TJA) is a successful orthopedic procedure that can significantly restore joint function, reduce ache, and enhance the standards of life among patients [1, 2]. However, it is predicted that by 2030, the ratio of total hip revisions will enhance by 137% and total knee revisions will enhance by 601% [3]. In a large sample study that included 35,140 patients from 2001 to 2010, Inacio et al. [4] found that initial instability, which accounted for 41.5% of cases, was the most common reason for revisions after total hip arthroplasty. In the National Joint Registry 12th Annual Report, among all reasons responsible for revision, aseptic loosening was the most frequent one, making up for 52.0% and 41.2% of 69,655 hip revisions and 36,722 knee revisions, respectively [5]. A recent study indicated that low bone mass around prostheses led to initial instability, aseptic loosening, periprosthetic fracture, and an increased rate of revisions [6]. Moreover, relevant problems associated with low bone mass around prostheses in osteoporosis patients are more obvious. Aro et al. [7] showed that postmenopausal osteoporosis patients with lower bone mineral density (BMD), which affects initial stability and delays stem osseointegration after TJA, had early prosthesis failure. Lacko et al. [8] showed that the speed of bone mass loss around prostheses was faster in postmenopausal osteoporosis than nonosteoporosis patients. Furthermore, a recent study showed that excessive oxidative stress exists in osteoporosis [9], which impairs bone remodeling, causing microstructural deterioration and bone mass loss [10]. To lengthen the lifespan of prostheses and decrease rates of implant failure, it is necessary to identify treatment strategies that protect bone mass around the prostheses in postmenopausal osteoporosis patients, thereby improving initial instability and aseptic loosening.

Melatonin is synthesized from serotonin in the human pineal gland and has many important effects, such as sleep induction, anti-inflammation, antitumor, and antioxidative properties [11–14]. Moreover, in recent years, the effects of melatonin on promoting bone formation have been increasingly reported and appear to differ from those of traditional antiosteoporosis drugs (bisphosphonates, denosumab, raloxifene, and teriparatide). Melatonin also has few adverse effects [15, 16], has a significant role in enhancing the growth of bone [17–20], and is cost-effective as a dietary supplement [21], making it an attractive candidate for further investigation. Additionally, the protecting function of melatonin against mitochondrial oxidative stress has been discovered among hepatocytes, cardiocytes, and oocytes [22–24]. Although Calvo-Guirado et al. [25] proved that melatonin can promote the osteointegration of dental implants, there have been no reports on whether it can improve bone mass around prostheses in osteoporosis, a condition that is linked to excessive oxidative stress.

The study is aimed at figuring out if melatonin can inhibit mitochondrial oxidative stress and improve bone mass around prostheses in osteoporosis.

## 2. Materials and Methods

### 2.1. Animals

All of the experiments had got the approval of the Ethics Committee of the First Affiliated Hospital of Soochow University (no. 201706A038), along with strictly adhered to guidelines for laboratory animal care and use. A total of 45 female 3-month-old Sprague-Dawley-specific pathogen-free rats were collected from the Experimental Animal Center of Soochow University and were adapted to the laboratory environment for 2 weeks before the experiment. The animals were placed in circumstance with constant temperature (23 ± 3°C) and humidity (45 ± 50%) and a 12-hour/12-hour light/dark cycle; what they need for eating and drinking were obtained at their own will. In the beginning of the experiment, the average weight of the rats was 250 ± 20 g.

### 2.2. Surgical Procedure

All of the animals received intraperitoneal (i.p.) injection of 10% chloral hydrate anesthesia (0.3 ml/100 g). Bilateral ovariectomy was performed in 30 rats, and sham operations were performed in the remaining 15 rats. After 3 months, BMD of all rats was measured using dual-energy X-ray absorptiometry (DEXA; Lunar Corporation, Madison, WI, USA) to assess whether the osteoporosis model had been successfully developed. Once the determination of success was made (Table 1), 45 rats underwent bilateral intramedullary titanium rod implantation. Titanium rods (15 mm in length, 1.5 mm in diameter, goodfellow titanium; Sigma, St. Louis, MO, USA) were sterilized and subjected to dual acid etching. The implant was put in the femoral canal of the rat. The 10 mm of the implant at the nearest end was pressed into the femoral bone marrow cavity for suitable preliminary fixation, along with the distal 5 mm of the implant was enclosed by a 0.25 mm gap. Then, 45 animals were classified into three groups at random: implant only (the sham group), ovariectomized (OVX) + implant (the control group), and OVX + implant + melatonin (the MT group); each group included 15 animals. The MT group received daily i.p. administration of melatonin at 50 mg/kg body weight (Sigma) between 4:00 p.m. and 6:00 p.m. for 4 weeks following implant installation. Melatonin was dissolved in 100% ethanol at a dilution of 50 mg/ml according to the protocol of manufacture and then diluted in sterile PBS. The sham group and control group were injected normal saline under the same conditions. In addition, all of the rats were injected i.p. 10 mg/kg calcein (Sigma) 10 and 2 days before euthanasia. In the last part of the experiment, the rats were sacrificed followed by the collection of their bilateral femurs for the subsequent studies.

### 2.3. Radiological Analyses

Femurs containing the titanium implants (*n* = 30 in each group) were measured with high-resolution microcomputed tomography (*μ*CT, SkyScan1176; SkyScan, Knotich, Belgium). The femurs were scanned with 18 *μ*m per layer, and the X-ray parameters were set at a voltage of 50 kV with a current of 500 *μ*A together with 0.7° rotational step. A related region of round shape with a diameter of 1.8 mm, located around one-third of the titanium rods of distal femurs, was chosen for the evaluation of related morphometric parameters including BMD (g/cm^3^), bone volume/total volume (BV/TV, %), bone surface/bone volume (BS/BV, 1/mm), trabecular number (Tb.N, 1/mm), trabecular thickness (Tb.Th, *μ*m), trabecular spacing (Tb.Sp, *μ*m), and connectivity density (Conn.D, 1/mm^3^). Relevant three-dimensional (3D) images were analyzed after processing.

### 2.4. Mechanical Pull-Out Test

All of the right femurs (*n* = 15 per group) were stored at -20°C for the mechanical pull-out test, which was performed after *μ*CT. A 10 mm distal resection was wrapped in dental cement to make each sample, using an X-ray imaging system as a guide. Then, the proximal position of the implant was located in every femur and the proximal implant (4-5 mm) was exposed after the cortical bone was removed. The exposed implant was clamped, and the fixture was mounted at the distant part of the specimen to make the force paralleled to the long axis of the implant. The pull-out test was performed with a displacement rate of 0.25 mm every minute. Peak load and elongation were recorded in the load-deformation curve.

### 2.5. Histological and Immunohistochemical Analyses

All of the left femurs (*n* = 15 per group) were preserved at 4°C for histology and immunohistochemical analyses after *μ*CT. Part of the left femur (*n* = 5 per group) was used for undecalcificated bone slicing, and slabs with a thickness of 1 mm were accomplished. Portions from 1 mm proximal to the tangent line of the femur growth plate were used for surveying of histomorphometry by light and fluorescence microscopy. The rest of the left femurs (*n* = 10 per group) were used for paraffin sections. The implant was carefully removed, and femur sections (with thickness of 5 *μ*m) were made for hematoxylin and eosin (H&E) staining to compare histological structures. Dynamic measurements involved single- and double-labeled perimeters, interlabel width, and bone mineral apposition rate (MAR), and static measurements included Tb.N and BV/TV. To identify the difference in the expression of osteogenetic markers, oxidative stress-related markers, and pathway-related markers in the abovementioned three groups, antibodies against the following were used for immunostaining: alkaline phosphatase (ALP, ab95462), osterix (ab22552), osteocalcin (OCN) (ab93876), NAPDH oxidase 2 (NOX2; ab80508), cytochrome c (cyto-c) (ab133504), sirtuin 3 (SIRT3; ab189860), and superoxide dismutase 2 (SOD2, ab13533) (all from Abcam, Cambridge, UK).

### 2.6. Cell Culture

MC3T3-E1 cells were cultured in alpha minimum essential medium (*α*-MEM) mineralizing medium (HyClone, Logan, UT, USA) including 10% fetal bovine serum and 1% penicillin-streptomycin in the situation with CO_2_ of 5% at 37°C. Hydrogen peroxide (H_2_O_2_) was purchased from Sigma, and SIRT3 small interfering RNA (siRNA) was synthesized by GenePharma (Shanghai, China). The cells were subsequently classified into five experimental groups: (1) the control group: cells were treated with *α*-MEM for 4 h; (2) the H_2_O_2_ group: cells were dealt with *α*-MEM added with H_2_O_2_ (400 *μ*M) for 4 h; (3) the H_2_O_2_ + MT group: cells were pretreated with *α*-MEM added with melatonin (100 *μ*M) for 1 h and consequently managed with *α*-MEM added with H_2_O_2_ (400 *μ*M) and melatonin (100 *μ*M) for 4 h; (4) the H_2_O_2_ + MT + SIRT3 siRNA group: cells were pretreated with SIRT3 siRNA for 4 h consequently, handled with *α*-MEM supplemented with melatonin (100 *μ*M) for 1 h and finally treated with *α*-MEM supplemented with H_2_O_2_ (400 *μ*M) and melatonin (100 *μ*M) for 4 h; and the (5) H_2_O_2_ + SIRT3 siRNA group: cells were pretreated with SIRT3 siRNA for 4 h and consequently treated with *α*-MEM supplemented with H_2_O_2_ (400 *μ*M) for 4 h.

### 2.7. Cell Viability Assay

Cell survival rate was measured with the Cell Counting Kit 8 (CCK-8) colorimetric assay (Dojindo, Rockville, MD, USA). Briefly, cells (5 × 10^3^) were cultivated in a 96-well plate. 10 *μ*l CCK-8 assay solution was supplemented to 100 *μ*l medium in every well, and we hatched the cells in the dark for two hours at 37°C before the cells were handled with different concentrations of H_2_O_2_ (0, 50, 100, 400, and 800 *μ*M) or melatonin (0, 25, 50, 100, 200, and 400 *μ*M) for 1, 3, and 5 days and washed with phosphate-buffered saline (PBS). Then, the optical density of every well was got on a microplate reader (SpectraMax M5; Molecular Devices, CA, USA). Cell survival rate was calculated by deciding the optical density ratio of cells in the experiment to that of control cells.

### 2.8. ALP Staining

One week after culture in osteogenic medium, the MC3T3-E1 cells were stained with ALP. Briefly, after the fixation of cells in 4% paraformaldehyde for 15 min, they were washed three times with PBS and consequently dipped in BCIP/NBT working solution in the dark for 20 min. The staining outcomes were obtained under a microscope.

### 2.9. Alizarin Red S Staining

Briefly, we washed MC3T3-E1 cells for three times with PBS after 3 weeks of incubation in osteogenic medium, followed by the fixation in 4% paraformaldehyde for 20 min at 4°C. Then, the cells were rinsed and incubated in alizarin red S (ARS) staining solution (pH 4.2; Cyagen Biosciences, Santa Clara, CA, USA) for 20 min. Finally, ddH_2_O was used to wash the cells three times.

### 2.10. Measurement of Reactive Oxygen Species

The cells were cultivated into 24-well plates with a density of 1 × 10^5^ cells/well for the whole day in three replicate wells. Then, the medium was replaced with *α*-MEM with or without H_2_O_2_ (400 *μ*M) or melatonin (100 *μ*M) for 4 h. Levels of reactive oxygen species (ROS) were surveyed employing the fluorescent probe 2′,7′-dichlorofluorescein diacetate (DCFH-DA) (Beyotime Institute of Biotechnology, Beijing, China). Briefly, DCFH-DA, diluted to an eventual density of 10 *μ*M, was added to the cells, which then were hatched for 20 min at a temperature of 37°C in the dark. The cells were measured by flow cytometry after being washed twice with PBS.

### 2.11. Real-Time PCR

We collected the total RNA applying TRIzol (Invitrogen). The total RNA density was surveyed by NanoDrop-2000 (Thermo Fisher, USA), for reverse transcription, 20 *μ*l per reaction using PrimeScript RT Master Mix (Takara, Japan), and PCR amplification, 20 *μ*l of each reaction, including 10 *μ*l of Forget-Me-Not qPCR Master Mix (Biotium, USA), 0.5 *μ*l of each primer, 2 *μ*l of cDNA, and 7 *μ*l RNase Free dH_2_O. The reaction was measured by the CFX96 Touch Real-Time PCR Detection System (Bio-Rad, USA). The cycle threshold values became the normal level of GAPDH. The following parts are primer sequences for ALP, OCN, osterix, and GAPDH: ALP forward 5′-CAGCGGGTAGGAAGCAGTTTC-3′ and reverse 5′-CCCTGCACCTCATCCCTGA-3′; OCN forward 5′-GAGGCTCTGAGAAGCATAAA-3′ and reverse 5′-AGGGCAATAAGGTAGTGAA-3′; osterix forward 5′-TGAGCTGGAACGTCACGTGC-3′ and reverse 5′-AAGAGGAGGCCAGCCAGACA-3′; and GAPDH forward 5′-GGTGAAGGTCGGTGTGAACG-3′ and reverse 5′-CTCGCTCCTGGAAGATGGTG-3′. Each sample was tested three times to reduce mistakes.

### 2.12. Western Blot

We seeded cells (1 × 10^6^ cells/well) in 6-well plates in triplicate wells for the whole day, and then the medium was replaced with *α*-MEM with or without H_2_O_2_ (400 *μ*M) or melatonin (100 *μ*M) or SIRT3 siRNA for 4 h. Proteins were obtained by centrifugation (12,000 rpm) for 15 min at 4°C. Protein samples were resolved by 15% sodium dodecyl sulfate polyacrylamide gel electrophoresis for 2 h along with electrophoretically transmitted to a polyvinylidene difluoride membrane (Millipore Corp., Bedford, MA, USA). After blocking nonspecific binding sites for 60 min in a situation where the temperature is the same as that in normal room with 5% skim milk, the membranes were hatched for the whole night at 4°C with primary antibodies against SIRT3 (1 : 1000), acetylated SOD2 (Ac-SOD2, 1 : 1000; ab137037), SOD2 (1 : 500), NOX2 (1 : 500), cyto-c (1 : 1000), Nrf2 (1 : 1000; ab62352), NAD(P)H: quinone oxidoreductase 1 (NQO 1, 1 : 1000; ab28947), ALP (1 : 1000), osterix (1 : 500), OCN (1 : 500), and *β*-actin (1 : 1000; ab8227). Then, membranes were rinsed in Tris-buffered saline with Tween 20 and incubated with corresponding secondary horseradish peroxidase-conjugated antibodies (1 : 1000) for 2 hours, a situation where the temperature is the same as that in normal room. The proteins were detected employing strengthened chemiluminescence reagents (Thermo Fisher Scientific, Waltham, MA, USA).

### 2.13. Statistical Analysis

Statistics are represented as the means ± standard deviations. The Kolmogorov-Smirnov test was employed to test statistics normality. The independent *t*-test and one-way analysis of variance were used for statistical analyses. SPSS 22.0 was employed for statistical analysis, and *P* < 0.05 was regarded to be with statistical difference.

## 3. Results

### 3.1. Melatonin Improved Periprosthetic Bone Mass and Increased Implant Fixation Strength in a Rat Model of Osteoporosis

**Figure 1:**
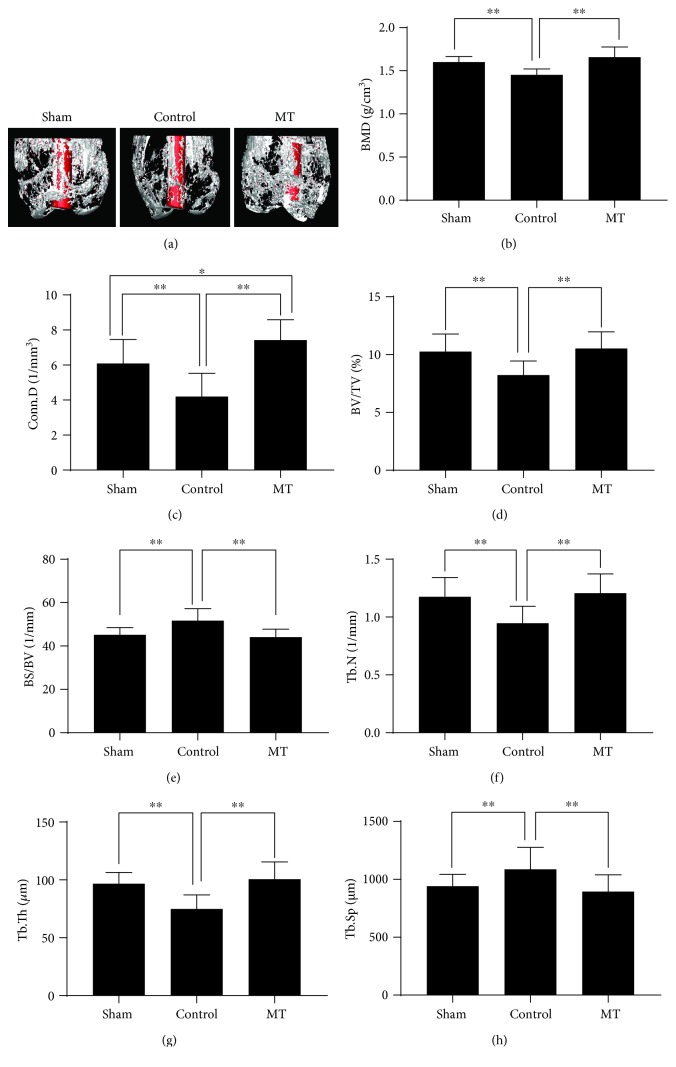
Melatonin treatment improved periprosthetic bone mass in a rat titanium rod-femur model. (a) 3D reconstruction of *μ*CT images. (b) BMD (g/cm^3^). (c) Conn.D (1/mm^3^). (d) BV/TV (%). (e) BS/BV (1/mm). (f) Tb.N (1/mm). (g) Tb.Th (*μ*m). (h) Tb.Sp (*μ*m). *n* = 30 per group. ^∗^*P* < 0.05 and ^∗∗^*P* < 0.01.

As shown in a three-dimensional reconstruction of *μ*CT images (Figure 1(a)), bilateral OVX caused a significant lower periprosthetic bone mass when compared with that in the sham group, whereas melatonin treatment greatly improved periprosthetic bone mass. In addition, data related to *μ*CT, BMD (1.658 ± 0.042 vs. 1.458 ± 0.024, g/cm^3^), BV/TV (10.53 ± 0.51 vs. 8.23 ± 0.43, %), BS/BV (44.12 ± 1.30 vs. 51.72 ± 1.96, %), Tb.N (1.21 ± 0.06 vs. 0.95 ± 0.05, 1/mm), Tb.Th (100.51 ± 5.32 vs. 74.82 ± 4.32, *μ*m), Tb.Sp (894.56 ± 51.22 vs. 1087.57 ± 67.25, *μ*m), and Conn.D (7.42 ± 0.41 vs. 4.19 ± 0.47, 1/mm^3^) in the MT and control groups showed that melatonin treatment significantly increased the periprosthetic bone mass in OVX rats (Figures 1(b)–1(h)).

**Figure 2:**
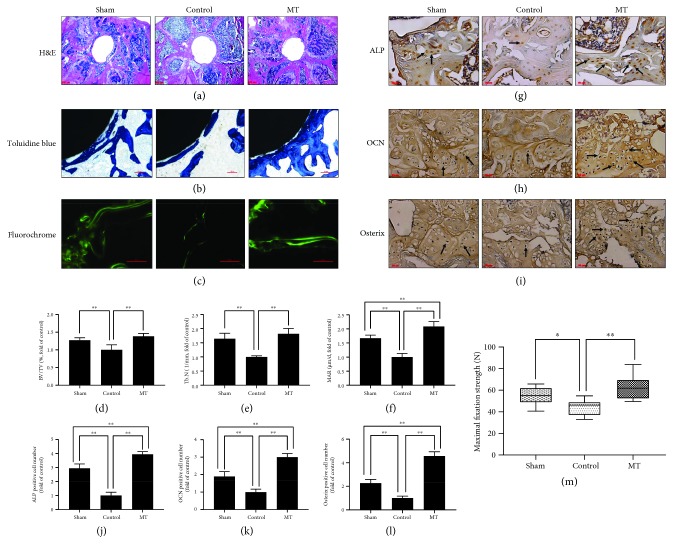
Melatonin treatment improved periprosthetic bone mass, promoted osteogenesis, and increased implant fixation strength. (a) H&E staining. Scale bar, 500 *μ*m. (b) Toluidine blue staining. Scale bar, 100 *μ*m. (c) High-magnification fluorochrome images. Scale bar, 100 *μ*m. (d) BV/TV (%, fold of control) from H&E staining, *n* = 10 per group. (e) Tb.N (1/mm, fold of control) from toluidine blue staining, *n* = 5 per group. (f) Mineral apposition rate (MAR) (*μ*m/d, fold of control) from high-magnification fluorochrome images, *n* = 5 per group. (g–l) Immunostaining of bone formation markers (ALP, OCN, and osterix). Scale bar, 50 *μ*m, *n* = 10 per group. (m) Mechanical pull-out test (maximal fixation strength, N), *n* = 15 per group. ^∗^*P* < 0.05 and ^∗∗^*P* < 0.01.

Consistent with the *μ*CT results, histological analysis further confirmed the bone protective effects of melatonin in osteoporosis rats. H&E staining (Figures 2(a) and 2(d)) and toluidine blue staining (Figures 2(b) and 2(e)) revealed that the amount of Tb around the prostheses significantly decreased in the control group when compared with the sham group, while melatonin significantly increased the amount of Tb around prostheses and BV/TV in the MT group compared with the control group. Single and double fluorochrome labeling, a direct histologic marker of bone formation, was more apparent and the distance between the double labels was greater in the sham group than in the control group, whereas melatonin treatment significantly improved single- and double-labeled perimeters, interlabel width, and MAR in the MT group compared with the control group (Figures 2(c) and 2(f)). Similarly, the ability of melatonin to improve osteogenesis was further confirmed by immunostaining. The expressing level of bone formation markers was decreased (ALP, OCN, and osterix) in the control group than in the sham group, whereas melatonin treatment clearly enhanced the expressing level of bone formation markers in the MT group when compared with the control group (Figures 2(g)–2(l)). The results of the mechanical pull-out test revealed that the maximal fixation strength in the MT group (62.85 ± 4.01 N) and sham group (54.79 ± 3.03 N) was greatly larger than that in the control group (44.05 ± 2.60 N; Figure 2(m)), which confirmed that melatonin can effectively increase implant fixation strength.

### 3.2. Melatonin Alleviated Oxidative Stress *In Vitro* and *In Vivo*

**Figure 3:**
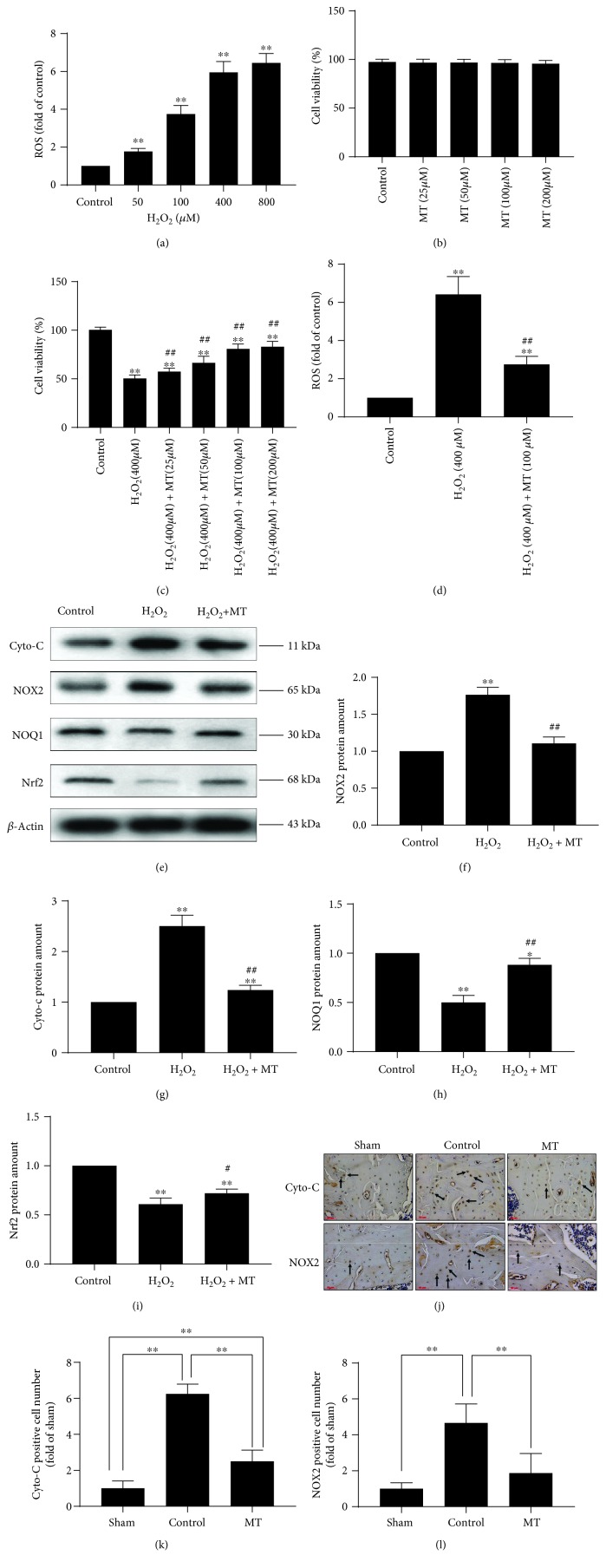
Melatonin alleviated mitochondrial oxidative stress *in vitro* and *in vivo*. (a) The correlation between different concentrations of H_2_O_2_ and ROS production (*n* = 3 per group, ^∗^*P* < 0.05 and ^∗∗^*P* < 0.01 vs. the control group). (b) The correlation among different concentrations of melatonin (0, 25, 50, 100, and 200 *μ*M) and cell viability without H_2_O_2_, *n* = 3 per group. (c) The correlation among different concentrations of melatonin (0, 25, 50, 100, and 200 *μ*M) and cell viability with H_2_O_2_ (400 *μ*M) (*n* = 3 per group, ^∗^*P* < 0.05 and ^∗∗^*P* < 0.01 vs. the control group, ^#^*P* < 0.05 and ^##^*P* < 0.01 vs. the H_2_O_2_ group). (d) Melatonin (100 *μ*M) alleviated oxidative stress in the H_2_O_2_ (400 *μ*M) model (*n* = 3 per group, ^∗^*P* < 0.05 and ^∗∗^*P* < 0.01 vs. the control group, ^#^*P* < 0.05 and ^##^*P* < 0.01 vs. the H_2_O_2_ group). (e–i) Western blot of cyto-c, NOX2, NOQ1, and Nrf2 (*n* = 3 per group, ^∗^*P* < 0.05 and ^∗∗^*P* < 0.01 vs. the control group, ^∗^*P* < 0.05 and ^∗∗^*P* < 0.01 vs. the H_2_O_2_ group). (j–l) Immunostaining of cyto-c and NOX2 (*n* = 10 per group, ^∗^*P* < 0.05 and ^∗∗^*P* < 0.01).

As shown in Figure 3(a), 400 *μ*M H_2_O_2_ significantly increased ROS levels; therefore, this concentration was used to create an oxidative stress model. As shown in Figures 3(b) and 3(c), melatonin had no toxicity in MC3T3-E1 cells and significantly increased the viability of MC3T3-E1 cells in the oxidative stress model. We also found that the effect was obvious when cells were treated with 100 *μ*M melatonin; therefore, this melatonin concentration was used for subsequent mechanistic studies. As expected, melatonin obviously decreased ROS levels when compared to that in H_2_O_2_-treated cells (Figure 3(d)). These results were confirmed by Western blot analysis (Figures 3(e)–3(i)).

NOX2 is a necessary part in NADPH-stimulated superoxide generation and leads to oxidative stress in pathological situation [26], whereas Nrf2 and NQO1 are two antioxidative molecules [27]. Compared with the control group, H_2_O_2_ significantly increased NOX2 protein amount and lowered Nrf2 and NQO1 protein amount. Conversely, melatonin downregulated NOX2 protein amount and upregulated Nrf2 and NQO1 protein amount, which confirmed the antioxidative effects of melatonin. Cyto-c, an important part of the electron transmission chain in mitochondria, clearly increases when the wholeness of mitochondrial is damaged [23]. The outcomes demonstrated that H_2_O_2_ significantly enhanced the levels of cyto-c, which were reduced after melatonin treatment, demonstrating that melatonin plays a part in protecting the wholeness of mitochondria. To verify our findings, immunostaining of NOX2 and cyto-c was performed (Figures 3(j)–3(l)). First, we found that two types of markers correlated with oxidative stress (NOX2 and cyto-c) dramatically increased in the control group when compared with the sham group, which proved that enhanced oxidative stress is present in osteoporotic rats. Second, we found that the expression of NOX2 and cyto-c clearly decreased in the MT group compared with the control group, consistent with our *in vitro* results and verifying the antioxidative impacts of melatonin *in vivo*.

### 3.3. The SIRT3/SOD2 Signaling Pathway Is Necessary and Significant in the Antimitochondrial Oxidative Stress Effects of Melatonin

**Figure 4:**
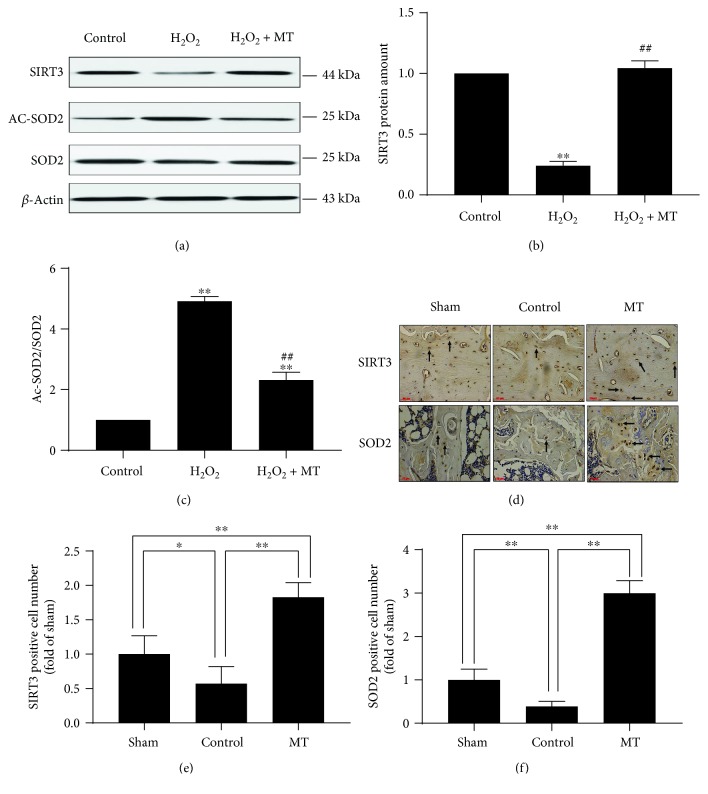
Melatonin alleviated mitochondrial oxidative stress via the SIRT3/SOD2 signaling pathway. (a–c) Western blot of SIRT3, Ac-SOD2, and SOD2 (*n* = 3 per group, ^∗^*P* < 0.05 and ^∗∗^*P* < 0.01 vs. the control group, ^#^*P* < 0.05 and ^##^*P* < 0.01 vs. the H_2_O_2_ group). (d–f) Immunostaining of SIRT3 and SOD2 (*n* = 10 per group, ^∗^*P* < 0.05 and ^∗∗^*P* < 0.01).

**Figure 5:**
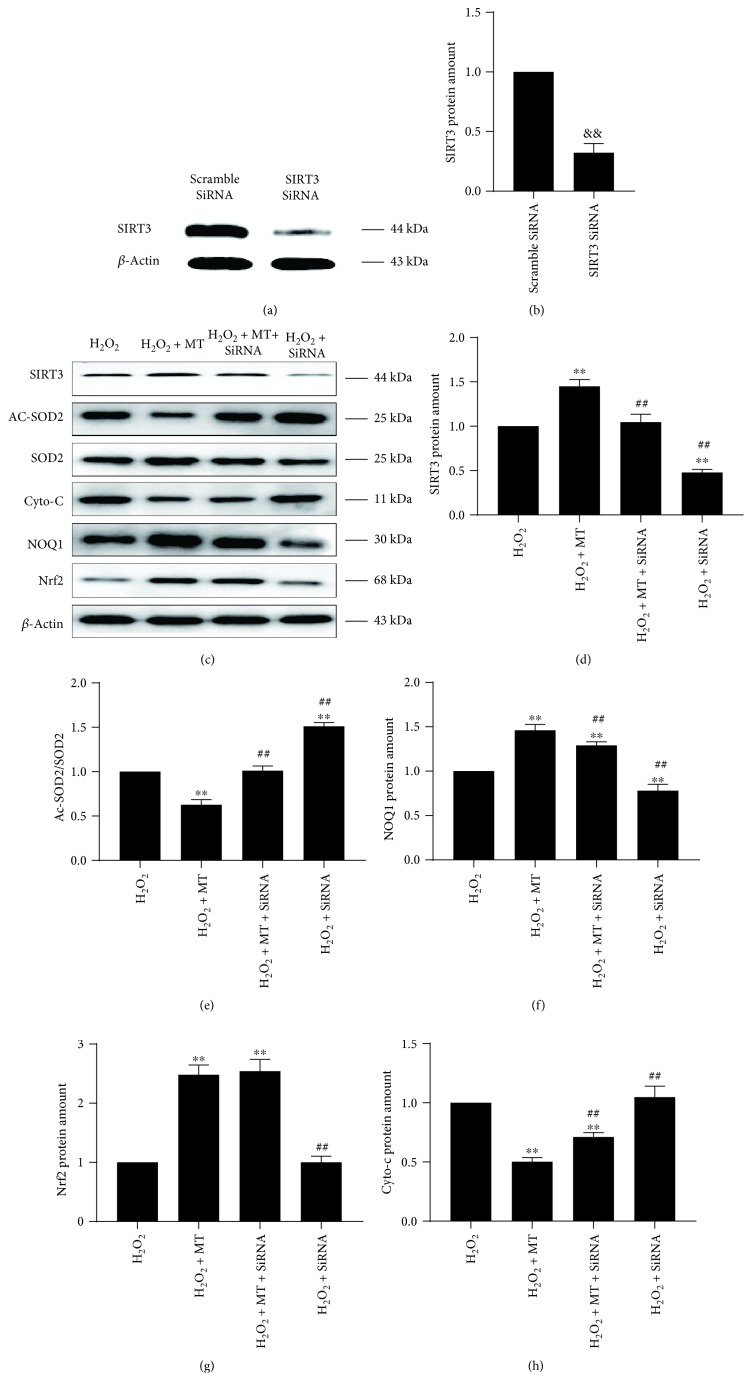
Activation of the SIRT3/SOD2 signaling pathway was essential to the antioxidative effects of melatonin. (a, b) The efficiency of SIRT3 siRNA was confirmed by Western blot (*n* = 3 per group, ^&&^*P* < 0.01 vs. the scramble SiRNA group). (c–h) Western blot of SIRT3, Ac-SOD2, SOD2, cyto-c, NOQ1, and Nrf2 (*n* = 3 per group, ^∗^*P* < 0.05 and ^∗∗^*P* < 0.01 vs. the H_2_O_2_ group, ^#^*P* < 0.05 and ^##^*P* < 0.01 vs. the H_2_O_2_ + MT group).

SIRT3 is primarily situated in mitochondria and is a significant member of the SIRT family, which has antioxidative effects that regulate SOD2 activity [24]. To determine if the SIRT3/SOD2 signaling pathway participates in the antioxidative effects of melatonin, the protein amount of SIRT3 and SOD2 was evaluated. As expected, H_2_O_2_ significantly decreased the protein amount of SIRT3 and increased the ratio of Ac-SOD2/SOD2, when compared with the control group. Conversely, melatonin noticeably increased SIRT3 protein amount and lowered the ratio of Ac-SOD2/SOD2 (Figures 4(a)–4(c)). In addition, the *in vivo* results showed that the expression of SIRT3 and SOD2 in the control and sham groups was lower than that in the MT group (Figures 4(d)–4(f)). These outcomes indicate that the antioxidative effects of melatonin may be through the SIRT3/SOD2 signaling pathway. We used SIRT3 siRNA to further determine whether the SIRT3/SOD2 signaling pathway modulates the antioxidative effects of melatonin. SIRT3 protein amount was significantly inhibited by SIRT3 siRNA (Figures 5(a)–5(d)). An increase in antioxidative activity induced by melatonin treatment, as shown by an increase in the protein amount of NQO1 and a decline in the Ac-SOD2/SOD2 ratio, was largely reversed by SIRT3 siRNA (Figures 5(c), 5(e) and 5(f)). There was little influence on the increase of Nrf2 protein amount by treatment with SIRT3 siRNA (Figures 5(c) and 5(g)). However, a decrease in cyto-c levels induced by melatonin was reversed by SIRT3 siRNA, which indicated that the ability of melatonin to protect the wholeness of mitochondria was impaired by SIRT3 siRNA (Figures 5(c) and 5(h)). Together, these results demonstrated that SIRT3/SOD2 activation was essential to the antioxidative effects of melatonin at the mitochondrial level.

### 3.4. Melatonin Alleviated Mitochondrial Oxidative Stress via the SIRT3/SOD2 Signaling Pathway to Promote Osteogenesis

**Figure 6:**
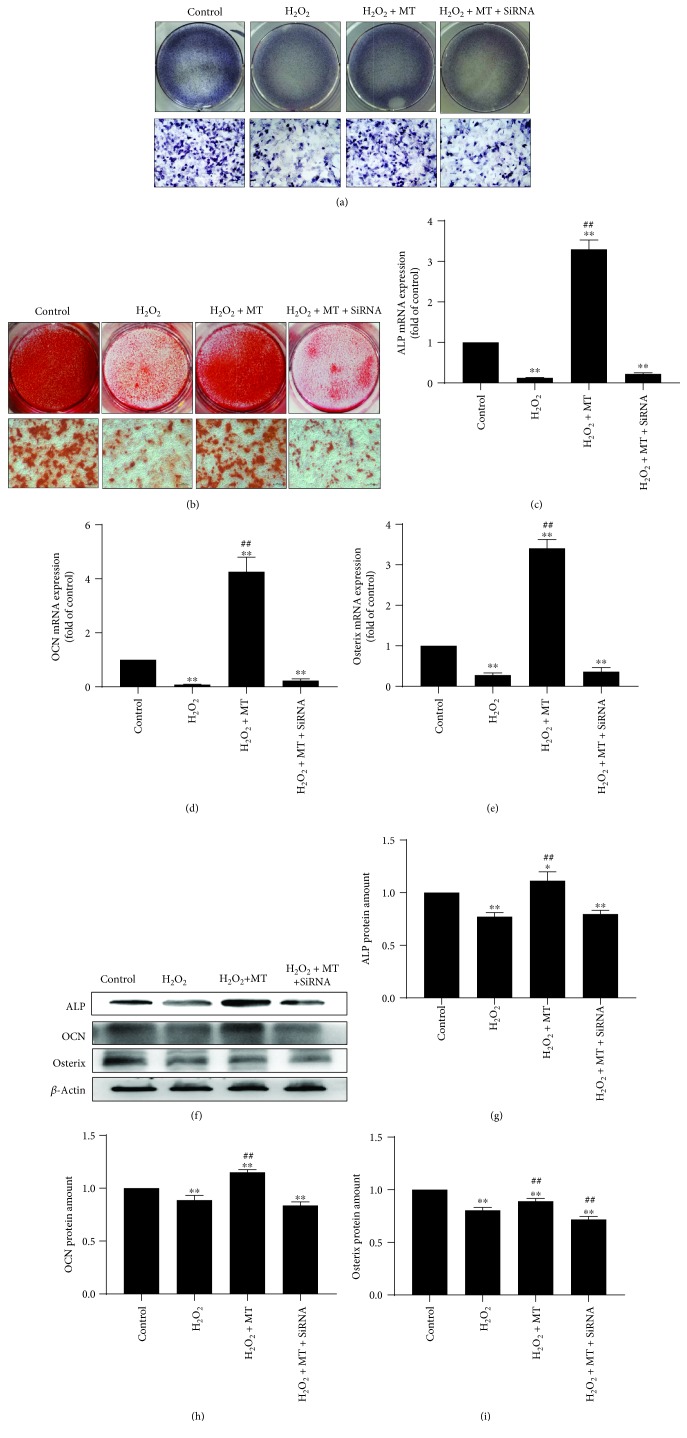
Positive correlation between effects of melatonin in promoting osteogenesis and alleviating mitochondrial oxidative stress. (a) ALP staining. (b) ARS staining. (c–e) RT-PCR of ALP, OCN, and osterix. (f–i) Western blot of ALP, OCN, and osterix. (*n* = 3 per group, ^∗^*P* < 0.05 and ^∗∗^*P* < 0.01 vs. the control group, ^#^*P* < 0.05 and ^##^*P* < 0.01 vs. the H_2_O_2_ group).

**Figure 7:**
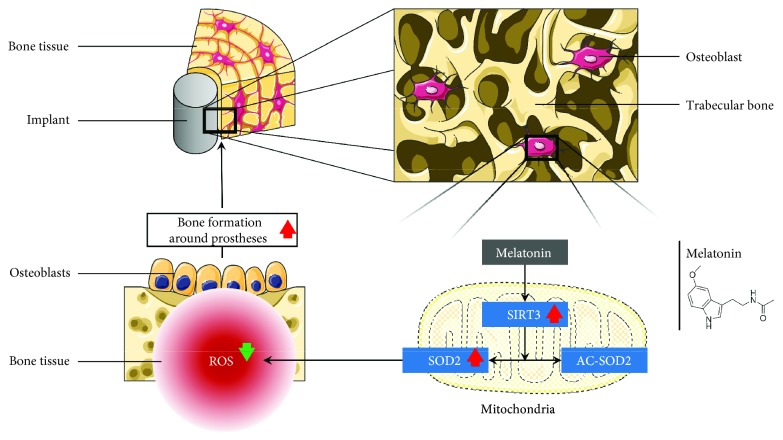
Melatonin ameliorates mitochondrial oxidative stress to improve bone mass around prostheses via the SIRT3/SOD2 signaling pathway.

We observed a positive correlation between the effects of melatonin on promoting osteogenesis and alleviating mitochondrial oxidative stress. Both ALP staining (Figure 6(a)) and ARS staining (Figure 6(b)) showed less osteogenesis in the H_2_O_2_ group when compared with the control group. Although melatonin treatment greatly enhanced osteogenesis in the MT group when compared to that in the H_2_O_2_ group, the use of SIRT3 siRNA reversed the effects of melatonin on osteogenesis. In addition, real-time PCR (RT-PCR) (Figures 6(c)–6(e)) and Western blotting (Figures 6(f)–6(i)) showed that expression of the bone formation markers OCN, ALP, and osterix decreased in the H_2_O_2_ group. After melatonin treatment, the expressing level of these markers significantly enhanced in the MT group compared with the H_2_O_2_ group; however, treatment with SIRT3 siRNA abolished these effects. These results confirmed that melatonin alleviated mitochondrial oxidative stress via the SIRT3/SOD2 signaling pathway to promote osteogenesis. The potential mechanisms of melatonin are showed in Figure 7.

## 4. Discussion

In recent years, the revision rates of TJA have rapidly increased, but the loss of bone mass around prostheses is the critical reason for implant failure, as it causes initial instability and aseptic loosening, especially in postmenopausal osteoporosis patients [3, 6, 7]. Unfortunately, there is no good solution for protecting bone mass around prostheses in osteoporosis patients. The traditional treatment protocols after TJA may be successful in inhibiting bone resorption, but few can promote osteogenesis, which is required in osteoporosis patients. In addition, the serious adverse effects of traditional treatment protocols, such as osteonecrosis of the jaw, subtrochanteric fractures, eczema, cellulitis, and osteosarcoma [28–35], have resulted in poor patient compliance. Melatonin is an excellent candidate for further investigation, as it has the excellent capacity to promote bone formation and few adverse effects [15–20]. Our study proved that melatonin ameliorates mitochondrial oxidative stress in osteoporosis, promotes osteogenesis, and increases bone mass around prostheses via the SIRT3/SOD2 signaling pathway.

Bone around prostheses undergoes intense remodeling after implant replacement, whereas most other bone serves as a relatively higher stage of development and the form of it is mineralized bone matrix, and peri-implanted bone has nonmineralized bone matrix [36, 37]. The osteointegration process not only remodels current bone but also enhances the formation of new bone around prostheses [38, 39]. Tresguerres et al. [40] showed that local melatonin application induced more trabecular bone at implant contact and higher trabecular area density. In this study, we showed that melatonin increased the generation of new bone around prostheses and stimulated the differentiation of osteoblasts, which is consistent with their results [40–42]. This increase in bone mass around prostheses after melatonin treatment may indicate greater synthesis of the peri-implant bone matrix, which may be related to the increase of osteoblast number or activity. According to some studies, melatonin promotes the proliferating and differentiating ability of human osteoblasts *in vitro*, and the length of time it takes for preosteoblasts to mature into osteoblasts is shortened after melatonin treatment [43–45]. In the study, the enhancement in osteoblast proliferation induced by melatonin was reflected.

Bone remodeling is a dynamic balanced process, in which homeostasis between bone resorption and bone formation is crucial [46]. The estrogen decline that occurs in postmenopausal women disrupts this homeostasis, causing an increase in ROS production in the bone microenvironment and degradation of the bone matrix [47, 48]. In osteoporosis, the increased production of ROS may largely contribute to the loss of bone mass around prostheses. Tan et al. [49] first showed melatonin as an antioxidant and free radical scavenger, and in recent years, the effects of melatonin in protecting against mitochondrial oxidative stress have been reported increasingly [22–24]. In this study, we found that melatonin promoted bone formation and ameliorated mitochondrial oxidative stress in osteoporosis. Thus, we gave more priority to the association between the bone-promoting and antimitochondrial oxidative effects of melatonin in osteoporosis.

First, we observed that melatonin inhibits mitochondrial oxidative stress via the SIRT3/SOD2 signaling pathway. Reiter et al. [50] showed that the melatonin concentration in other parts of the cell is significantly lower than that in mitochondria, so melatonin should be divided into a mitochondria-targeted antioxidant. SIRT3, a greatly conserved nicotinamide adenine dinucleotide-dependent deacetylase localized in the mitochondria, is regarded as the most significant acetyl-lysine deacetylase that modulates varied proteins to regulate mitochondrial effects along with ROS production [51]. Therefore, we suspected a correlation between melatonin and SIRT3. In addition, SOD2 is the main target of SIRT3, and the effects of mitochondrial oxidative stress have been extensively proven [52]. SIRT3 eliminates ROS, controlling ROS homeostasis by transforming AC-SOD2 into SOD2 [52–55]. In our model of oxidative stress, we watched a clear decrease in SIRT3 expression and an increase in the Ac-SOD2/SOD2 ratio in MC3T3-E1 osteoblast precursor cells. When compared with the H_2_O_2_ group, melatonin increased SIRT3 protein amount and decline of the ratio of Ac-SOD2/SOD2. These results confirmed the effects of melatonin in protecting against mitochondrial oxidative stress via the SIRT3/SOD2 signaling pathway.

Second, the essentiality of the SIRT3/SOD2 signaling pathway in the antimitochondrial oxidative stress effects of melatonin and whether there is a positive correlation between these effects and promoting osteogenesis should be defined. Normal mitochondrial function and homeostasis of mitochondrial biogenesis are critical for osteogenesis. ROS causes mitochondrial dysfunction and impairs biogenesis, which is a fundamental pathological process that inhibits osteogenesis [56–59]. SIRT3 is located in the mitochondria, with the capacity to regulate ROS homeostasis and maintain mitochondrial function and biogenesis to preserve osteogenesis [60]. SOD2 plays an important role in mediating the effects of SIRT3 on ROS [61, 62]. In this study, we observed that the antimitochondrial oxidative stress capacity of melatonin significantly decreased with SIRT3 siRNA treatment. In addition, the ability of melatonin to promote osteogenesis was also decreased with SIRT3 siRNA treatment. These results showed that the SIRT3/SOD2 signaling pathway is a significant and necessary part in the antimitochondrial oxidative effects of melatonin and that antimitochondrial oxidative stress and promoting osteogenesis were positively correlated.

## 5. Conclusion

In summary, our study showed that melatonin ameliorates mitochondrial oxidative stress to improve bone mass around prostheses in osteoporosis via the SIRT3/SOD2 signaling pathway. Our outcomes revealed that melatonin treatment is potentially important for patients after TJA, especially patients with osteoporosis. Additional studies are needed to confirm our observations.

## Figures and Tables

**Table 1:** Effects of ovariectomized (OVX) on bone mineral density (BMD) after 3 months of surgery (g/cm^2^).

Group	Lumbar vertebrae	Femur	Whole body
Sham	0.187 ± 0.002	0.142 ± 0.003	0.171 ± 0.002
OVX	0.179±0.001^∗∗^	0.129±0.002^∗∗^	0.160±0.002^∗∗^

BMD of OVX rats was significantly reduced compared with sham rats after 3 months of surgery (15 rats in the sham group and 30 rats in the OVX group, ^∗^*P* < 0.05 and ^∗∗^*P* < 0.01 vs. the sham group).

## Data Availability

The data used to support the findings of this study are available from the corresponding author upon request.
